# Correlation of thermal Doppler flowmetry and microdialysis values in patients with severe subarachnoid hemorrhage and traumatic brain injury

**DOI:** 10.1186/cc13647

**Published:** 2014-03-17

**Authors:** DC Papadopoulos, P Papamichalis, As Filippidis, D Karangelis, TH Bekiari, KN Fountas, G Vretzakis, KN Paterakis, A Komnos

**Affiliations:** 1 General Hospital of Larisa, Greece; 2 Boston Medical Center, Boston, MA, USA; 3 Onassis Cardiac Surgey Center, Athens, Greece; 4 Prefectural Authority of Thessaly, Larisa, Greece; 5 University Hospital of Larissa, School of Medicine, University of Thessaly, Larisa, Greece

## Introduction

The purpose of this study is to investigate the relationship between continuously monitored regional cerebral blood flow (CBF) and microdialysis values in severe subarachnoid hemorrhage and traumatic brain injury patients.

## Methods

Advanced multimodal neuromonitoring including measurements of CBF (QFlow, Hemedex) and brain lactate, pyruvate, lactate/ pyruvate ratio, glycerol and glucose values using microdialysis (CMA600, microdialysis) were performed in 21 patients with severe subarachnoid hemorrhage (*n *= 17) and traumatic brain injury (*n *= 4). Thirteen of the patients were successfully discharged from the ICU while eight did not survive. Additional recorded parameters include PbrO_2 _(Licox, GMS) ICP, CPP, MABP, CVP, local brain temperature, body core temperature, PCO_2_, and blood glucose among others. The cerebral monitoring probes were inserted via a Bolt (ICP, PbrO_2_, microdialysis) and an additional burr hole (CBF). All probes were positioned in the penumbra and location was verified by brain CT. The PbrO_2 _arm of this study and its significance is still underway and will be announced later. Thirteen of the patients were successfully discharged from the ICU while eight did not survive.

## Results

The final data are currently under statistical evaluation, which will be completed at the time of presentation. However, there is indication of a link between brain glucose levels and CBF values, but it is not clear as to the CBF-PbrO_2 _correlation that is the second part of this study under evaluation. This may be due to the fluctuation of brain glucose because of brain ischemia, hyperemia, hypermetabolism or hypometabolism. So far we are able to establish a correlation of CBF and lactate/pyruvate ratio only in persistently low CBF values.

## Conclusion

This will be a final report of a study in human patients with severe subarachnoid hemorrhage and traumatic brain injury. The results indicate correlations of varying significance between the pooled data still under statistical analysis. We hope that the outcome of our study will be able to answer questions regarding the pathophysiology of severe brain injury and guide us in the titration of therapy, as it is needed by each individual patient [1-4].
